# Retentive Forces and Deformation of Fitting Surface in RPD Clasp Made of Polyether-Ether-Ketone (PEEK)

**DOI:** 10.3390/polym15040956

**Published:** 2023-02-15

**Authors:** Sunil Kumar Vaddamanu, Fahad Hussain Alhamoudi, Saurabh Chaturvedi, Nasser M. Alqahtani, Mohamed Khaled Addas, Mohammad Al Alfarsi, Rajesh Vyas, Masroor Ahmed Kanji, Mohammad A. Zarbah, Waleed M. S. Alqahtani, Saeed M. Alqahtani, Adel M. Abdelmonem, Asim Elsir Elmahdi

**Affiliations:** 1 Department of Dental Technology, College of Applied Medical Sciences, King Khalid University, Abha 61421, Saudi Arabia; 2 Department of Prosthetic Dentistry, College of Dentistry, King Khalid University, Abha 61421, Saudi Arabia

**Keywords:** polyether-ether-ketone (PEEK), Co-Cr, RPD, circumferential clasp, deformation

## Abstract

Background: Polyetheretherketone (PEEK) has provided the option to fabricate RPDs with aesthetics unlike metal RPDs, but little attention has been paid to its suitability, especially towards the retentive forces and deformation of the clasp. This study aimed to examine the retentive forces and the fitting surface (inner surface) deformation of clasps made from PEEK and compare it with cobalt–chromium (Co-Cr) clasp. Methods: Forty-two circumferential clasps (14 Co-Cr and 28 PEEK) were fabricated and divided into two groups with clasp undercuts (0.25 mm and 0.5 mm) with thicknesses of 1 mm and 1.5 mm. Each was examined for retentive forces after cycle test on its abutment for 360 cycles. Initial and final retentive forces were recorded. The fitting surface deformation was determined using 3-Matic research analysis software. Results: The results revealed that highest mean initial retentive force was of Co-Cr clasps with 0.50 mm undercut 22.26 N (±10.15 N), and the lowest was the 1 mm PEEK clasps with 0.25 mm undercut 3.35 N (±0.72 N) and highest mean final retentive force was the Co-Cr clasps with 0.50 mm undercut 21.40 N (±9.66 N), and the lowest was the 1 mm PEEK clasps with 0.25 mm undercut 2.71 N (±0.47 N). PEEK clasps had a lower retentive force than Co-Cr clasps with 0.50 undercut. PEEK clasps (1.5 mm) at 0.25 mm undercut had the least deformation (35.3 µm). PEEK showed significantly less deformation (*p* ≤ 0.014) than Co-Cr. Conclusion: The deformation of PEEK clasps fitting surface was lower than Co-Cr clasps and retentive forces were close to the Co-Cr clasps, suggesting the use of PEEK as an aesthetic clasp option for RPD framework.

## 1. Introduction

Replacement of missing teeth with a fixed prosthesis or implants is the preferred choice of any patient, but affordability and anatomical or systemic health reasons are sometimes confounding factors. In such cases, removable dental prostheses are used for temporary and long-term oral rehabilitation in fully and partially edentulous subjects [1]. At present, metal alloys such as cobalt-chromium (Co-Cr) and titanium are commonly used by manufacturers of removable dental prostheses because of their high strength and stiffness and corrosion resistance [2]. Conversely, metal alloys have the drawbacks of being unaesthetic, having a potential risk of metallic taste, and causing allergies. The main purpose of RPDs or any dental prosthesis is to maintain oral function by restoring missing teeth and surrounding tissues while maintaining the patient’s appearance, comfort, and health [3]. This point enhances the demand for a material that is biocompatible, aesthetic and has adequate strength when used for RPD fabrication [4,5,6,7,8,9].

Conventionally, RPDs are fabricated with Co-Cr alloys or titanium alloys [4], which creates aesthetic issues. Various methods have been suggested in the literature to overcome this problem. For example, Lammie and Osborne [6] described the mesiodistal clasp that engages the mesial and distal surfaces of the tooth. The Equipoise clasp [7] similarly engages the proximal tooth surface. King et al. suggested the use of lingual retentive clasps, and Highton et al. proposed the use of palatal I bars or dual paths or rotational paths of insertion [8,9]. Others have attempted to camouflage the visible clasps by covering them with tooth-colored veneers [9,10,11,12,13], while removable partial dentures with precision attachments are aesthetically satisfying, but they are expensive and more technique-sensitive [14].

With the advancement in the field of materials and the rapid evolution of computer-aided design and computer-aided manufacturing (CAD/CAM) technology, polyetheretherketone (PEEK), a high-performance thermoplastic semi-crystalline polymer, has gained popularity. PEEK is a promising alternative material for metal-free removable dental prostheses that presents favorable characteristics such as superior mechanical properties, good thermal and chemical resistance, and radiographic radiolucency. Various studies have been conducted on the effectiveness of the clasps and their effect on the abutment teeth [15,16,17]. A clasp arm should produce less stress on the abutment, be flexible, and return to its original form. Generally, metals and metal alloys undergo permanent deformation and fatigue when exposed to repeated stress, which is an important consideration in the metal selection for RPD fabrication [18]. In a previous study, authors used constant deflection tests to investigate clasp fatigue and concluded that clasp fatigue affected retentive properties and that loss of retention may be caused by the permanent deformation of clasps [19]. Various research studies have been conducted on the properties of materials used for removable partial denture clasps, but very few studies have been found on PEEK [15,16,17,18,19].

In recent years, free-metal prostheses have been preferred by both dentists and patients because of their biocompatibility and aesthetic benefits. Therefore, polyether-ether-ketone (PEEK) has been introduced in dentistry as a restorative material for fabricating fixed and removable prostheses [20,21]. PEEK is a member of the poly-aryl-ether-ketone (PAEK) family of high-performance thermoplastic polymers used in a variety of industrial applications due to their chemical structure, physical stability, and high melting temperature [22]. Although some studies have been carried out on the effectiveness of such materials as dental restorations, little attention has been given to the suitability of PEEK as RPD.

Thus, the present study was conducted with the aim to examine the retentive forces and the fitting surface (inner surface) deformation of clasps made from PEEK and compare them with cobalt–chromium (Co-Cr) clasps using 3D software after the cycle test. The null hypothesis formulated was that there would be no difference in the assessed properties of the clasps made from PEEK and Co-Cr.

## 2. Materials and Methods

### 2.1. Materials

The present study was conducted at King Khalid University, Abha, KSA, in the Department of Prosthodontics, College of Dentistry and an ethical waiver was granted by the institute’s ethical committee since it was an in-vitro study without the involvement of bodily tissues. Two groups (Gr) were made based on the materials used for clasp fabrication—Gr-1- Clasps made from cobalt-chromium (Co-Cr) (Wironit, Bego, Bremen, Germany) as a control group; Gr-2- Clasps made from polyether-ether-ketone (PEEK) (BreCAM.BioHPP, Bredent, Chesterfield, UK), as the test group.

### 2.2. Methodology

**Figure 1:**
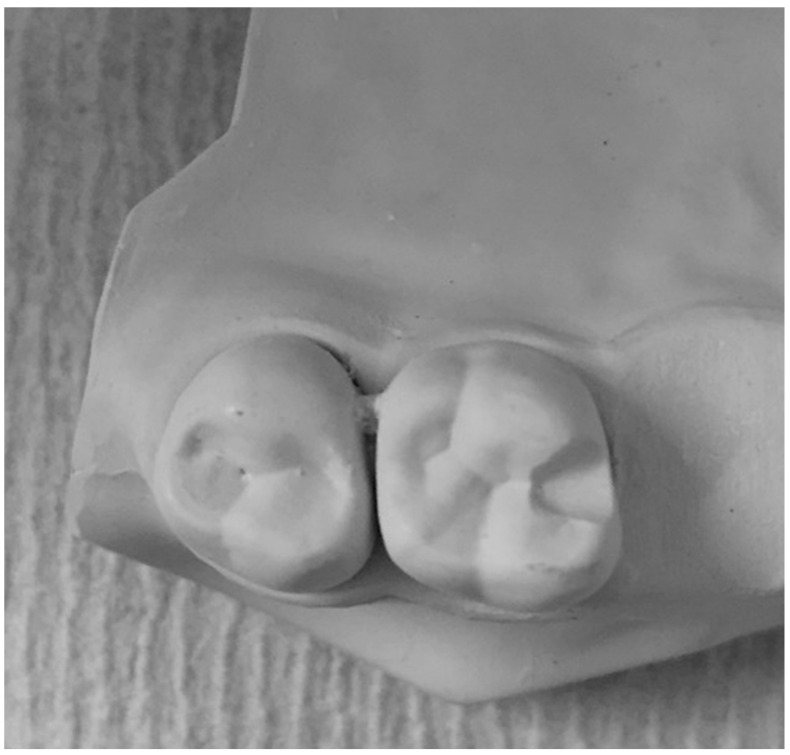
Representative image of maxillary second molar model prepared with mesial occlusal rest seat.

For the fabrication of study samples, two maxillary jaw teeth set dental stone model was used. The maxillary second molar (Dentona^®^ Esthetic-Base^®^ gold, Dentona AG, Dortmund, Germany) was prepared in the model (the first molar was cut) to receive a circumferential clasp with the desired undercut (Figure 1). The maxillary jaw model was surveyed and an undercut of 0.25 mm on one model and 0.50 mm on the other were created. Occlusal rests (2.5 mm long, 2.5 mm wide, and 2 mm deep) were placed mesially. The prepared molar models were duplicated with silicon impression material (Dublisil, Dreve Den-tamid GmbH, Unna, Germany). The impressions were poured into resin material (RHINO ROOCK, DB Lab Supplies Limited) to fabricate 42 molars models: 21 with a 0.25 mm undercut and 21 with a 0.50 mm undercut [23].

### 2.3. Clasp Designing

To standardize the position, shape, size, and thickness of the clasps, a CAD/CAM system was used. The molar models were scanned, using a 3D scanner (Identica Blue, Medit, 19 Inchon-RO 22-GIL, Seongbuk-GU, Seoul, Republic of Korea). Four clasps were designed using the CAM design system (Dental Wing Operating System, CAD-DWOS RPD design V–5.2.2).

- Two 3D Circumferential (Conventional (Long Arm)) clasps (1.0 mm thick with occlusal rests, retentive and reciprocal arms) were designed to engage in an undercut of 0.25 mm on one clasp and 0.50 mm on the other. In the base design, the clasps with the 0.25 mm undercut had a wide base whereas the clasps with the 0.50 mm undercut had a narrow base to differentiate between them.
- Two 3D short arm clasps (1.5 mm thick with occlusal rests, retentive and reciprocal arms) were designed to engage in an undercut of 0.25 mm on one clasp and 0.50 mm on the other. The previously described base designs were applied here, but the wide base was used for the 0.50 mm and the narrow one for the 0.25 mm (Table 1).

Each design had a base and holder, parallel to the path of insertion. Each clasp had three support pins to keep it in position during milling.

### 2.4. Co-Cr Clasps Fabrications

**Figure 2:**
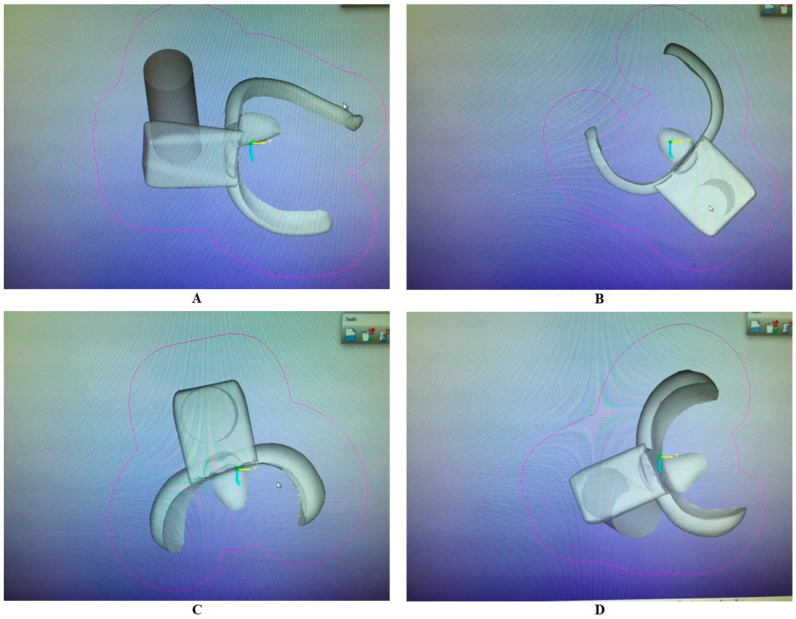
(**A**) Circumferential clasp design 0.50 (narrow base), (**B**) circumferential clasp design 0.25 (wide base), (**C**) short arm clasp design 0.50 (wide base), and (**D**) short arm clasp design 0.25 (narrow base).

The CAD file (. Stl form) was used to fabricate the clasps via the computer-aided manufacturing production method (CAM) (Figure 2A–D).

A Roland milling machine (DWX–50 Roland, Roland DG (UK) Ltd., North Somerset, UK) was used to mill seven circumferential clasps (1 mm thickness) for each undercut (0.25 mm and 0.50 mm) from a wax blank (14 mm thick, 98 mm diameter) (Professional Milling Wax, BRISTOL CADCAM). Fourteen wax clasps were fabricated. A round wax sprue, 4 mm thick (Wachsprofile, Bego), was connected to the meeting point between the base and holder to avoid any casting failure. The wax clasps were then invested and cast in Co-Cr alloy with an induction casting machine using high-frequency induction melting technology. Co-Cr clasps were disinvested, and sprues were eliminated. To ensure uniformity of the clasps, finishing and polishing were performed with the identical methodology for every clasp using a stone bur. In total, 14 Co-Cr clasps were produced.

### 2.5. PEEK Clasp Fabrication

**Figure 3:**
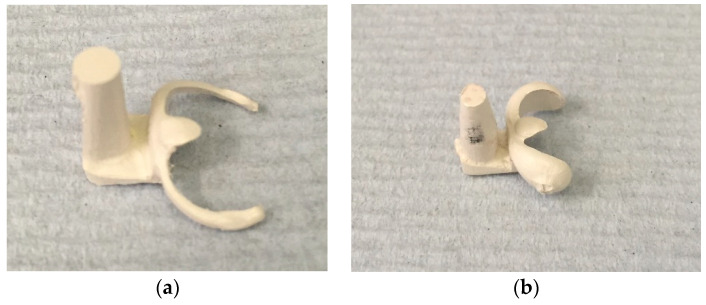
(**a**) Circumferential PEEK clasp and (**b**) short arm PEEK clasp.

The CAD file (.stl form) was used to fabricate 28 PEEK clasps via the CAM. A Roland milling machine (DWX-50 Roland, Roland DG (UK) Ltd., Clevedon, UK) was used. Seven circumferential clasps (1 mm thick) of each undercut (0.25 mm and 0.50 mm) and seven short arm clasps (1.5 mm thick) of each undercut (0.25 mm and 0.50 mm) were fabricated from a PEEK blank (16 mm thick, 98 mm diameter) (BreCAM.BioHPP, Bredent, Chesterfield, UK) (Figure 3a,b).

### 2.6. Retentive Forces and Cycling Test Analysis

**Figure 4:**
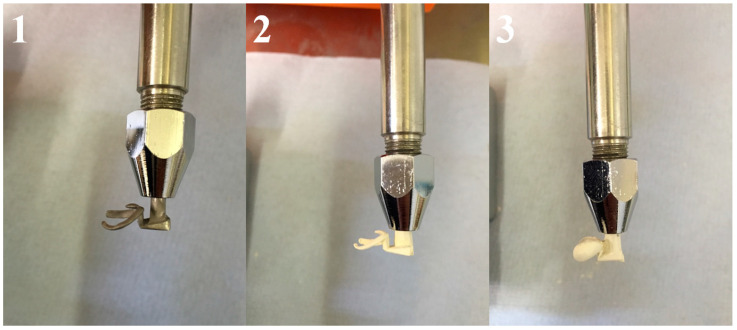
(**1**) Circumferential Co-Cr clasp, (**2**) circumferential PEEK clasp, and (**3**) circumferential PEEK clasp.

PEEK and Co-Cr clasps were evaluated under a cycling test simulating six months of use, using a fatigue chewing simulator machine tensile tester unit (2.5 KN Lloyd LRX, West Sussex, UK). The machine allowed the placement of the clasp to its predetermined terminal position and its subsequent removal from the resin molar, thus simulating the placement and removal of an RPD. The resin models (RHINO ROOCK, DB Lab Supplies Limited, Silsden, UK) were fixed to the lower part of the masticatory simulator. Each clasp specimen was then located on the corresponding resin molar and fixed to the upper part of the machine with a metallic holder (Figure 4).

**Figure 5:**
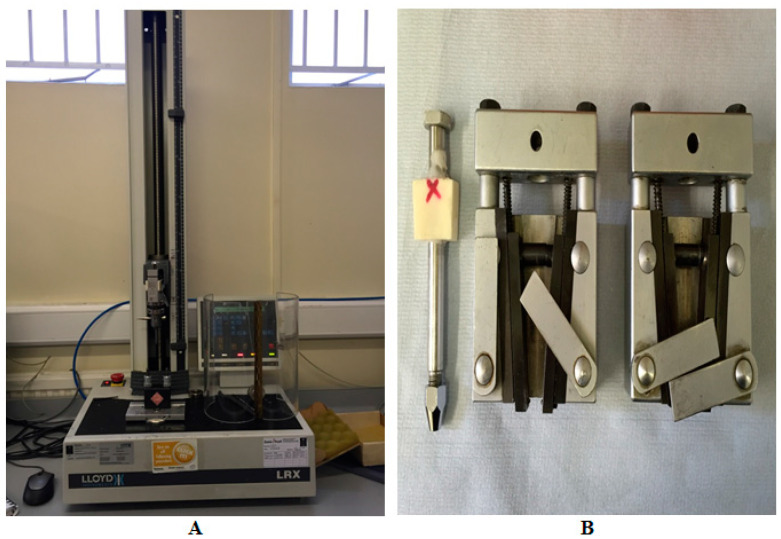
(**A**) Fatigue chewing simulator machine. (**B**) From left to right: metallic holder, upper and lower part.

**Figure 6:**
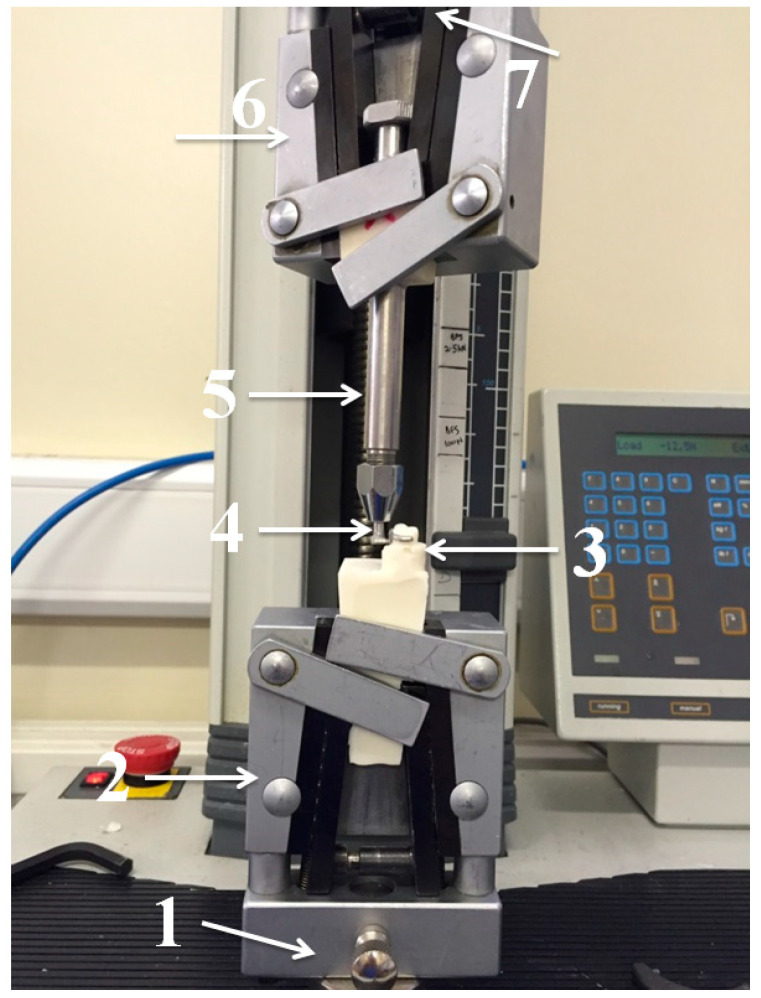
Cycling test. (**1**) Lower position; (**2**) lower part; (**3**) abutment crown with holder; (**4**) clasp with holder; (**5**) metallic holder; (**6**) upper part; and (**7**) upper position.

The test conditions were maintained at room temperature (20 ± 2 °C). To analyze the data obtained during the simulation test, a total of 360 cycles were established and performed. This represented the simulated insertion and removal of the RPD over six months, estimating the patient would perform two removals and insertions of the RPD per day. The test was conducted at a constant speed of 240 mm/m with the distance of removal and insertion being 5 mm, representing 360 cycles of 15 min for each clasp. The force required for each specimen removal was captured as retentive force and stored using data acquisition software (NEXYGENPlus software, AMETEK Sensors, Test and Calibration, Lloyd Materials Testing, West Sussex, UK). (Figure 5 and Figure 6).

### 2.7. Fitting Surface (Inner Surface) Deformation Analysis

PEEK and Co-Cr clasps were evaluated under a cycling test simulating six months of use, using a fatigue chewing simulator machine tensile tester unit (2.5 KN Lloyd LRX). The test conditions were maintained at room temperature (20 ± 2 °C). During the simulation test, a total of 360 cycles were established and performed. This represented the simulated insertion and removal of the RPD over six months, estimating the patient would perform two removals and insertions of the PRD per day. The test was conducted at a constant speed of 240 mm/m with the distance of removal and insertion being 5 mm, representing 360 cycles of 15 min for each clasp.

In preparing the samples for scanning, Co-Cr clasps could not be scanned due to their shiny surface, which reflected on the scanner light. Therefore, blasting material of 50 µm alumina (Korox 50, Bego) at 0.25 MPa pressure was applied to the Co-Cr clasp to create a scannable surface. For PEEK clasps, no preparation was performed, since it is a scannable material. Before and after the cycle test, the fitting surface (inner surface) of both the Co-Cr and PEEK clasps were scanned using a 3D Scanner (Identica Blue, Medit, 19 Inchon-RO 22-GIL, Seoul, Republic of Korea) to provide CAD files (. Stl form) [23,24,25,26].

**Figure 7:**
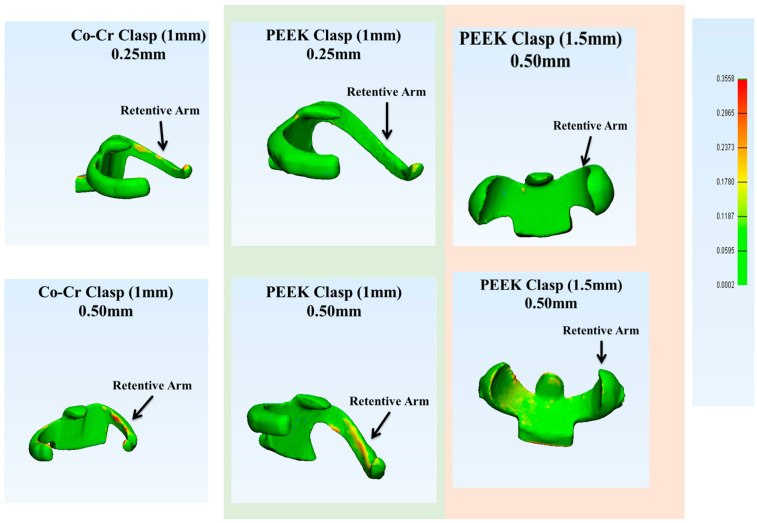
From the right, the standard reading was fixed from 0.0002 mm (**green color**) to 0.3558 mm (**red color**). The upper part of the photo shows the clasps with 0.25 mm; the lower part shows clasps with 0.50 mm undercut.

In the present study, the 3D analysis software (3-matic^®^ Software, Materialise, Technologielaan 15, 3001 Leuven, Belgium) was used as it had the advantages of producing and using CAD files over conventionally used methods such as a universal testing machine. It helped in analyzing the deformation and reducing human errors. Then the 3D analysis software (3-matic^®^ Software, Materialise, Technologielaan 15 Leuven, Belgium) evaluated the deformation of clasps’ fitting surface (inner surfaces) using part comparison analysis, where the lowest number meant low deformation, and the highest meant high deformation. After the analysis, the initial histogram ranges were different. Therefore, the lowest and highest readings were recorded (0.0002 mm–0.3558 mm) and applied to all samples to standardize the range for analysis (Figure 7). For statistical analysis purposes, the data were converted from millimeter (mm) to micrometer (µm) (1 mm = 1000 µm). The statistical analysis was done with one-way ANOVA using Statistical Package for the Social Sciences (SPSS). The significance level was fixed at 5% (α = 0.05).

## 3. Results

### 3.1. Retentive Forces and Cycling Test

Table 2 and Table 3 show the mean initial and final retentive forces (after 360 cycles) for all subgroups during the test. The group with the highest mean initial retentive force was the Co-Cr clasps with 0.50 mm undercut 22.26 N (±10.15 N) and the lowest was the 1 mm PEEK clasps with 0.25 mm undercut 3.35 N (±0.72 N); the group with highest mean final retentive force was the Co-Cr clasps with 0.50 mm undercut 21.40 N (±9.66 N) and the lowest was the 1 mm PEEK clasps with 0.25 mm undercut 2.71 N (±0.47 N).

**Figure 8:**
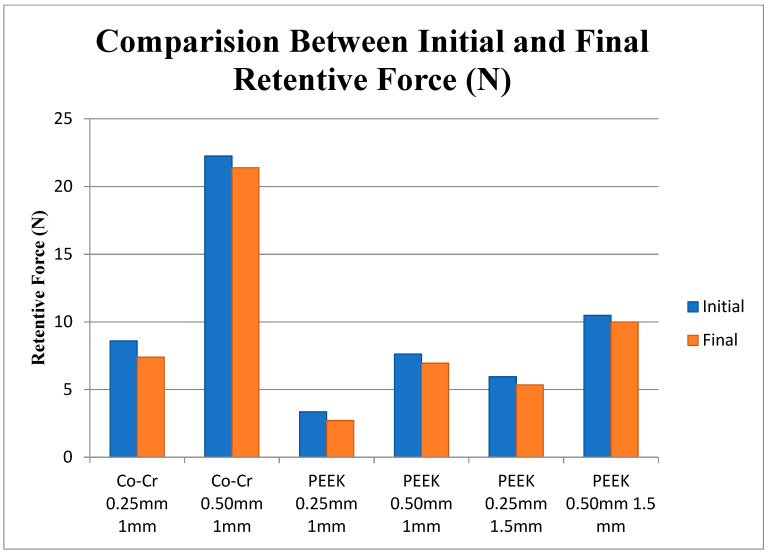
Comparison between initial and final retentive force (N).

Figure 8 present the changes in retentive force required to remove clasps from the 0.25 mm and 0.50 mm undercuts before and after the test. All the clasps exhibited a decrease in retentive forces at the end of the cycle test from approximately 0.50 to 1 N. Overall, all the clasps with 0.50 mm undercut produced a high retentive force compared to the clasps with 0.25 mm. Clearly, the Co-Cr clasps with 0.50 mm undercut produce the highest retentive force, due to the low flexibility of Co-Cr.

### 3.2. Fitting Surface (Inner Surface) Deformation Analysis

**Figure 9:**
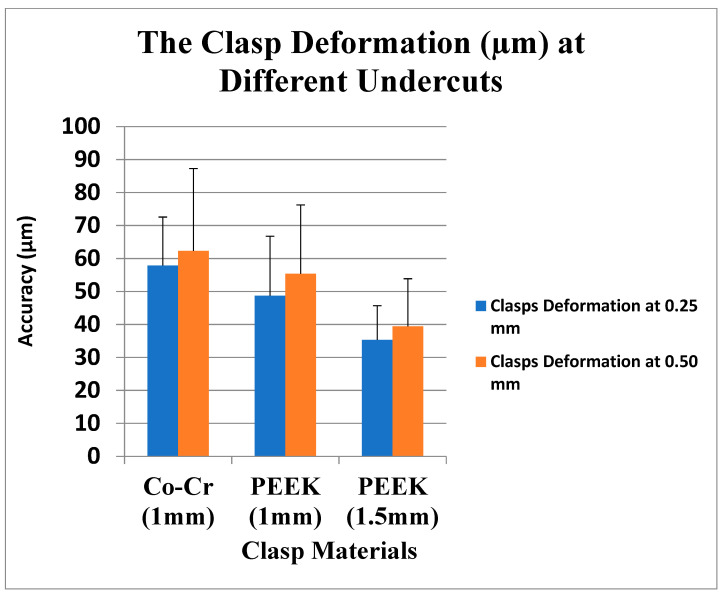
The deformation of clasps inner surfaces (µm).

The study revealed that there were deformations or changes in the retentive arms of all clasps. Furthermore, clasps with 0.25 mm undercut showed fewer changes than those with 0.50 mm undercut. The deformation range for all clasps of the Co-Cr clasps at 0.25 mm undercut was from 42.5 to 85.6 µm, and at the 0.50 mm undercut it was from 46.6 to 90.5 µm. The 1 mm PEEK clasps at 0.25 mm were from 28.8 to 84.7 µm, and the 0.50 mm undercut were from 28.3 to 79.5 µm. The 1.5 mm PEEK clasps at 0.25 mm were from 17.9 to 50.3 µm, and 0.50 mm were from 23.6 to 68.4 µm. The least deformation clasps were the 1.5 mm PEEK clasps with 0.25 mm undercut (35.29 µm (±10.41)), and the highest deformation clasps were the Co-Cr clasps with 0.50 mm undercut (62.27 µm (±15.56)). Overall, the fitting surface of all clasps with 0.50 mm undercut presented higher deformation than the clasps with 0.25 mm (Table 4 and Figure 9).

The one-way ANOVA test revealed that PEEK showed significantly less deformation (*p* ≤ 0.014) than Co-Cr. In design, the 1.5 mm clasp design had significantly less deformation (*p* ≤ 0.002) than Co-Cr. In the undercut, the clasps with 0.25 mm undercut had less deformation than Co-Cr, but this was insignificant (*p* = 405) (Table 5 and Table 6). At 0.25 mm undercut, the material type showed a significant effect. PEEK clasps showed less deformation than Co-Cr at 0.50 mm undercut, and the material type showed no significant difference (Table 7).

Therefore, the deformation comparisons with the Tukey multiple tests determined no significant change between Co-Cr 1 mm and PEKK 1 mm nor between PEEK (1 mm and 1.5 mm) (*p* > 0.05). However, there was a significant difference between Co-Cr 1 mm and PEEK 1.5 mm (*p* ≤ 0.026) (Table 8). All the clasps exhibited better fitting surfaces at 0.25 than those at 0.50 mm, but it was still not significantly different (*p* > 0.05).

## 4. Discussion

Conventional RPDs have an inherent problem of an unaesthetic appearance and metal allergies. This has resulted in demand for metal-free RPDs. Various studies have been conducted using PEEK as a potential replacement for metal RPD clasps. However, retention and clasp deformation has not been established. Based on the present research results, the PEEK claps showed significantly less deformation on the fitting surface (inner surface), particularly the 1.5 mm clasps, than the Co-Cr. Consequently, the null hypothesis that there would be no difference in the fitting surface deformation between PEEK clasps and Co-Cr alloy clasps is rejected.

### 4.1. Retentive Forces and Cycle Test

One of the most important qualities of clasps is their retention force, which will resist the removable prosthesis’ dislodging force during masticatory function and muscle movement. When assessing the fit of a clasp material to the retentive area, the clasp shape (length, width), flexibility, and undercut engagement should be considered [27]. The optimum performance of a clasp depends on the balance between these variables. The results of the present study showed that PEEK clasps of both dimensions (1 mm and 1.5 mm) with both undercuts (0.25 mm and 0.50 mm) had lower retentive force than Co-Cr clasps. However, Sato et al. and others [28,29,30,31,32] suggested that the retentive force from 3 to 7.5 N is expected to have adequate retention for RPD. In this study, the retentive force at the end of the cycling test ranged, for the 1.0 mm PEEK clasps, from 1.91 to 10.51 N, and for the 1.5 mm thick clasps from 3.82 to 13.63 N. Accordingly, PEEK could be applied as clasps for RPD, as it provides adequate retention for RPD, even after six months of simulated usage. Being a soft and ductile material, PEEK can yield nicely and adapt well [33]. Polyetheretherketone and PEKK are modifications of the main thermoplastic high-performance polymer group, polyaryletherketone (PAEK) [34,35]. Polyetheretherketone has high thermal and chemical stability as it has an aromatic backbone molecular chain, interconnected by ketone and ether functional groups. Polyetherketoneketone is a liner thermoplastic polymer and consists of a benzene ring attached consecutively by ether or ketone groups. The second ketone group in PEKK ensures better mechanical and physical properties [36]. Polyetherketoneketone has higher compressive strength, excellent polishing ability, high biocompatibility, good mechanical properties, high-temperature resistance, highly polished surface, low plaque affinity, and acceptable low specific weight [34].

The Co-Cr clasps in this study with a 0.50 mm undercut provided the highest retentive force, due to the low flexibility of CoCr. According to De Torres et al. and Rodrigues et al. [3,33], the retentive arms of Co-Cr clasps are recommended to be engaged at a 0.25 mm undercut to give affordable retentive force on the abutment. Fitton et al. [30] reported that thermoplastic clasps must have a greater cross-sectional diameter than metal clasps to provide suitable retention. Additionally, Turner et al. [31] suggested a clasp design for thermoplastic materials where clasp arms must be around 5 mm long with a large cross-section diameter (1.4 mm) to produce stiffness similar to that of a Co-Cr clasp with arms 15 mm long and 1 mm in width. Furthermore, the undercut of the thermoplastic clasp improves the retentive force, which was reported by Tannous et al. [32]. In this current study, the greatest retentive force for PEEK clasps was found in the 1.5 mm thick clasps designed with 0.50 mm undercut (Initial = 14.53 N; Final = 13.63 N), which could be similar to the 1 mm Co-Cr clasps with 0.25 mm undercut (Initial = 12.63 N; Final = 11.56 N). Hence, PEEK should be engaged at a deep undercut (0.50 or 0.75 mm) and be thicker than metal clasps to achieve clinically acceptable retention. This is because of the low elastic modulus (rigidity) of PEEK (3–4 GPa for PEKK as opposed to 211–228 GPa for the Co-Cr alloy) [37,38,39,40,41]. An essential element affecting the clinical durability of removable prosthesis clasps is fatigue resistance. Clasps undergo repeated bending caused by mastication and by the insertion and removal of the removable prosthesis, which may cause fatigue failure during long-term use [42]. PEEK has a low elastic modulus (3.0–4.0 GPa), which plays an important role in fatigue testing [43]. Additionally, the flexibility and ductility of PEKK result in a good fit and mechanical adaptation.

Furthermore, there was not a huge difference between the initial and the final retentive forces. This could be due to the rigid system of the cycle test, where the clasps were fixed in the upper part and abutment to the low part of the test machine. This method ensured a straight path of insertion and removal and eliminated any possibilities of torquing during the test, which may have affected the test results positively. In a patient’s mouth or under clinical conditions, the results might be affected negatively due to different insertion and removal paths, based on the anatomical aspects of teeth and the mobility of natural teeth. Furthermore, patients can change the path of insertion and/or removal used to move the RPD creating greater loads on the tooth, which may lead to clasp deflection or fracture in a short period [44,45,46,47,48,49]. Consequently, more studies on clinical conditions are required.

### 4.2. Fitting Surface (Inner Surface) Deformation

In this current project, the 3-matic analysis system was used to examine the fitting surface of PEEK clasps and compare them to Co-Cr. The critical analysis of such a system is that it can integrate both scanned inner surfaces, and then compare them as one unit. Specifically, it indicates the mini differences or deformations in µm. The greatest reading shows high deformation, and the lowest number shows low deformation.

The results showed that the inner surfaces of all clasps had excellent matching after the cycle test. However, the most significant deformations were in the retentive arms where the majority of the forces were applied. In this study, PEEK clasps of both dimensions (1 mm and 1.5 mm) with both undercuts (0.25 mm and 0.50 mm) had lower readings in the retentive arms than the Co-Cr clasps. The reading range of deformations was from 28.3 to 84.7 µm for 1 mm PEEK clasps, and from 17.9 to 68.4 µm for 1.5 mm PEEK. The Co-Cr was from 42.5 to 90.5 µm. Therefore, PEEK could be used as clasps for RPD as it causes less deformation to the clasps’ inner surface than Co-Cr for RPD, even after six months of simulated usage.

The great retentive force of Co-Cr clasps with 0.50 mm undercut negatively affects its inner surface as it showed the highest reading (90.5 µm) and mean (62.3 µm) of deformation. However, the PEEK clasps in both dimensions and undercuts exhibited less inner surface deformation than the Co-Cr clasps. Even though the PEEK 1.5 mm clasps with 0.50 mm undercut had retentive forces that could be similar to the Co-Cr ones with 0.25 mm, there was significantly less deformation in the fitting surface of PEEK than Co-Cr, particularly the 1.5 mm clasps with 0.25 mm (*p* ≤ 0.026). The low deformation of PEEK clasps’ inner surfaces may produce adequate retention for a long period, and eliminate or reduce any possibility of creating marks on the abutment surface as reported by Zoidis et al. [24].

As noted above, PEEK clasps showed less deformation than Co-Cr. In the comparison between PEEK clasps, the 1 mm with both undercuts (0.25 and 0.50 mm) exhibited higher deformations than the 1.5 mm clasps. This was not expected due to the high retentive force of the 1.5 mm PEEK clasps. The logical explanation of such a case is that the 1 mm PEEK clasps are affected by clasp design factors. The low cross-section, high curve, and long and thin clasp arms, in addition to the brittle nature of PEEK, create clasps that are easily deformed [26,36,39]. Accordingly, PEEK clasps are recommended to be engaged at a deep undercut, such as 0.50 mm, and be thicker than metal clasps to achieve acceptable stiffness and high resistance to deformation [28,29,30].

In this study, the results of the 3-matic analysis could have been affected by the pre-scan procedures. In comparison, the PEEK clasps were a scannable material, but the Co-Cr clasps were not, due to their shiny surface after the polishing and finishing procedures. As a result, the Co-Cr clasps were blasted with 50 m alumina at 0.25 MPa pressure to create a scannable surface. These producers might cause further deformation to the inner surface of Co-Cr clasp, and this could affect the results negatively. Accordingly, more studies on the Co-Cr surface are needed.

## 5. Conclusions

It is widely considered that polyether-ether-ketone (PEEK) is a biocompatible material, with exceptional mechanical properties. It has the potential to be a restorative material in dentistry. The literature review provided earlier presented evidence for the aesthetic, biocompatible, and mechanical properties of this material compared to metal alloys, particularly Co-Cr. Nevertheless, before this study, it was unknown whether PEEK clasps could provide lesser or higher deformation resistance compared to Co-Cr clasps. The availability of certain data in this field, such as those described here, is critical to inform the scientific debate regarding the quality of PEEK clasps. From the analysis of the results presented, the following conclusions can be drawn.

1. The mean of Co-Cr clasps retentive force was higher than PEEK clasps. However, the 1.5 mm PEEK clasps with a 0.50 mm undercut showed a retentive force close to that of the Co-Cr clasps with 0.25 mm undercut.
2. The clasps with 0.25 undercut showed lower deformation in clasps fitting surface (inner surface) than those with 0.50 mm.
3. The deformation of all PEEK clasps’ fitting surfaces (inner surface) was lower than that of Co-Cr clasps.
4. The deformation of 1.5 mm PEEK clasps with 0.25 undercut inner surface was significantly lower (*p* ≤ 0.026) than the Co-Cr clasps, but no significant change was found between the 1.0 mm and 1.5 mm PEEK clasps.
5. The 1.5 mm PEEK clasps with both undercuts (0.25 mm and 0.50) showed the highest deformation resistance, even though they exhibited a retentive force similar to Co-Cr. Accordingly, this design is recommended for use with PEEK clasps.

## Figures and Tables

**Table 1:** The clasps’ design and sample distribution.

Design	Undercut	Thickness	Base	n* (PEEK)	n* (Co-Cr)
Circumferential Clasp with Long Arm	0.25	1 mm	Wide	7	7
	0.50	1 mm	Narrow	7	7
Circumferential Clasp with Short Arm	0.25	1.5 mm	Narrow	7	–
	0.50	1.5 mm	Wide	7	–

* Total n = 42 (n-Peek = 14/14; n-Co-Cr = 14).

**Table 2:** The initial retentive forces (N).

Sample	Co-Cr 0.25 mm 1 mm	Co-Cr 0.50 mm 1 mm	PEEK 0.25 mm 1 mm	PEEK 0.50 mm 1 mm	PEEK 0.25 mm 1.5 mm	PEEK 0.50 mm 1.5 mm
1	9.11	12.34	4.75	10.65	4.96	12.08
2	6.41	9.19	3.70	5.69	5.92	14.53
3	13.78	34.64	2.68	8.51	4.39	7.40
4	8.33	14.93	3.01	6.80	4.82	10.57
5	4.11	31.39	2.91	7.79	5.20	11.58
6	5.77	23.54	2.87	6.45	8.75	9.88
7	12.63	29.76	3.56	7.50	7.62	7.31
Mean	8.59	22.26	3.35	7.63	5.95	10.48
S.D.	3.57	10.15	0.72	1.62	1.63	2.58

**Table 3:** The final retentive forces (N).

Sample	Co-Cr 0.25 mm 1 mm	Co-Cr 0.50 mm 1 mm	PEEK 0.25 mm 1 mm	PEEK 0.50 mm 1 mm	PEEK 0.25 mm 1.5 mm	PEEK 0.50 mm 1.5 mm
1	8.98	12.34	4.21	10.51	4.63	11.57
2	6.22	8.61	2.53	5.85	4.74	13.63
3	9.95	32.15	2.15	7.03	3.84	6.45
4	5.93	13.97	2.87	5.29	3.82	10.77
5	3.91	29.39	2.80	6.85	4.81	11.22
6	5.16	23.18	1.91	6.45	8.41	9.32
7	11.56	30.17	2.49	6.66	7.19	7.01
Mean	7.39	21.40	2.71	6.95	5.35	10.00
S.D.	2.80	9.66	0.47	1.68	1.76	2.57

**Table 4:** The mean deformation of clasps (µm).

Undercut	Co-Cr (1 mm)	PEEK (1 mm)	PEEK (1.5 mm)
0.25 mm	57.9	48.7	35.3
0.5 mm	62.3	55.3	39.4

**Table 5:** ANOVA test of material effect.

Source	Type III Sum of Squares	df	Mean Square	F	Sig.	Partial Eta Squared
Corrected Model	2206.313 ^a^	1	2206.313	6.628	0.014	0.142
Intercept	102417.567	1	102417.567	307.686	0.000	0.885
Material	2206.313	1	2206.313	6.628	0.014	0.142
Error	13314.559	40	332.864			
Total	119742.320	42				
Corrected Total	15520.871	41				
^a^. R Squared = 0.142						

**Table 6:** ANOVA test of undercut effect.

Source	Type III Sum of Squares	df	Mean Square	F	Sig.	Partial Eta Squared
Corrected Model	269.547 ^a^	1	269.547	0.707	0.405	0.017
Intercept	104221.449	1	104221.449	273.344	0.000	0.872
Undercut	269.547	1	269.547	0.707	0.405	0.017
Error	15251.325	40	381.283			
Total	119742.320	42				
Corrected Total	15520.871	41				
^a^. R Squared = 0.017 (Adjusted R Squared = –0.007)						

**Table 7:** ANOVA test of material effect 0.25 mm and 0.50 mm.

Source	Type III Sum of Squares	df	Mean Square	F	Sig.
Corrected Model	1804.287 ^a^	2	902.143	4.162	0.033
Intercept	46945.258	1	46945.258	216.582	0.000
Material	1804.287	2	902.143	4.162	0.033
Error	3901.586	18	216.755		
Total	52651.130	21			
Corrected Total	5705.872	20			
^a^. R Squared = 0.316 (Adjusted R Squared = 0.240)					
Corrected Model	1920.487 ^a^	2	960.243	2.267	0.132
Intercept	57545.738	1	57545.738	135.846	0.000
Material	1920.487	2	960.243	2.267	0.132
Error	7624.966	18	423.609		
Total	67091.190	21			
Corrected Total	9545.452	20			
^a^. R Squared = 0.201 (Adjusted R Squared = 0.112)					

**Table 8:** Tukey multiple tests of clasps material at 0.25 mm and at 0.50 mm.

(I) Material	(J) Material	Mean Difference (I–J)	Std. Error	Sig.	95% Confidence Interval	
					Lower Bound	Upper Bound
Co-Cr-1 mm-0.25	PEEK-1 mm-0.25	9.1571	7.86956	0.489	−10.9273	29.2415
	PEEK-1.5 mm-0.25	22.5714 *	7.86956	0.026	2.4870	42.6558
PEEK-1 mm-0.25	Co-Cr-1 mm-0.25	−9.1571	7.86956	0.489	−29.2415	10.9273
	PEEK-1.5 mm-0.25	13.4143	7.86956	0.231	−6.6701	33.4987
PEEK-1.5 mm-0.25	Co-Cr-1 mm-0.25	−22.5714 *	7.86956	0.026	−42.6558	−2.4870
	PEEK-1 mm-0.25	−13.4143	7.86956	0.231	−33.4987	6.6701
Co-Cr-1 mm-0.50	PEEK-1 mm-0.50	6.9286	11.00142	0.806	−21.1488	35.0060
	PEEK-1.5 mm-0.50	22.8429	11.00142	0.123	−5.2346	50.9203
PEEK-1 mm-0.50	Co-Cr-1 mm-0.50	−6.9286	11.00142	0.806	−35.0060	21.1488
	PEEK-1.5 mm-0.50	15.9143	11.00142	0.339	−12.1631	43.9917
PEEK-1.5 mm-0.50	Co-Cr-1 mm-0.50	−22.8429	11.00142	0.123	−50.9203	5.2346
	PEEK-1 mm-0.50	−15.9143	11.00142	0.339	−43.9917	12.1631

*. The mean difference is significant at the 0.05 level.

## Data Availability

Data can be made available on demand by the chief researcher for academic purposes by email.
